# A Lecithin-Based Delivery Form of Quercetin Promotes Stress Resistance and Longevity in *Caenorhabditis elegans*

**DOI:** 10.3390/ph19040525

**Published:** 2026-03-24

**Authors:** Margherita Romeo, Maria Monica Barzago, Claudia Fracasso, Manuel Nettis, Antonella Riva, Marco Gobbi, Serena Tongiani, Luisa Diomede

**Affiliations:** 1 Department of Molecular Biochemistry and Pharmacology, Istituto di Ricerche Farmacologiche Mario Negri IRCCS, Via Mario Negri 2, 20156 Milan, Italy; mariamonica.barzago@marionegri.it (M.M.B.); claudia.fracasso@marionegri.it (C.F.); manuel.nettis@marionegri.it (M.N.); marco.gobbi@marionegri.it (M.G.); 2 Product Portfolio Office, Indena SpA, 20139 Milan, Italy; antonella.riva@indena.com (A.R.); serena.tongiani@indena.com (S.T.)

**Keywords:** quercetin, lecithin-based quercetin formulation, phytosome, *C. elegans*, aging, stress

## Abstract

**Background/Objectives**: The flavonoid quercetin (Q) has recently been suggested as a natural anti-aging and senolytic agent. However, its low stability and poor oral bioavailability may limit its efficacy. To address this, we investigated whether a lecithin-based formulation of Q, Quercefit™ (QF), enhances stress resistance and delays aging in vivo. **Methods**: The nematode *Caenorhabditis elegans* was used as an animal model to evaluate the effects of QF under physiological and stress conditions. Unformulated Q was administered as a control. Worm survival, healthspan, resistance to thermal and oxidative stress, and expression of stress- and longevity-related genes were assessed. All the experiments were conducted at least in triplicate, each including a minimum of 15 worms. The data were analyzed using Student’s *t*-test, one-way or two-way ANOVA, and Bonferroni’s post hoc test. **Results**: One hundred micromolar Q administered in QF was more effective than equimolar unformulated Q in increasing the worms’ ability to resist acute thermal stress at 35 °C (tested on 75 worms/group) and oxidative stress caused by 0.5 mM hydrogen peroxide (tested on 75 worms/group). In this last case, the protective effect of QF was similar to that of N-acetylcysteine and ascorbic acid. Under experimental conditions mimicking the long-term consequences of thermal stress, QF, like Q, increased the worms’ lifespan and healthspan by approximately 50%, counteracting the age-related decline associated with stress (120 worms/group). These benefits are supported by QF’s capacity to act as a reactive oxygen species scavenger; suppress heat-shock element gene transcription activated by thermal stress, such as *hsp-16.2* and *hsp-70*, and stimulate the *sod-3* and *gst-4* genes that are involved in antioxidant and detoxification responses. **Conclusions**: These findings suggest that Q, when administered in the QF formulation, can act at the transcriptional level to protect against aging induced by stressful conditions.

## 1. Introduction

Aging is linked to a progressive loss of cellular homeostasis, accompanied by increased oxidative burden and cellular damage, accumulation of senescent cells, and reduced stress resistance, contributing to greater susceptibility to age-related diseases [1,2,3,4].

Polyphenolic flavonoids, which are widely distributed in plants, are recognized for their significant biological activities, including protection against oxidative stress, inflammation, and atherosclerosis in various model organisms [5]. Among flavonoids, quercetin (3,3′,4′,5,7-pentahydroxyflavone) (Q), which is abundant in vegetables and fruits, is often regarded as the most important flavonoids in dietary and medicinal contexts. Its intake has been associated with positive health effects due to its antioxidant and free-radical-scavenging properties [6].

Studies on the nematode *Caenorhabditis* (*C.*) *elegans* demonstrated that Q can positively affect longevity by extending the worms’ lifespan and healthspan [7,8] and increasing their resistance to thermal and oxidative stress [9]. These effects may be partially attributed to the flavonoid’s ability to modulate the insulin/insulin-like growth factor signaling (IIS) pathway. This important, evolutionarily conserved pathway contains components with high homology to human aging-related genes [10]. Q was reported to reduce IIS pathway activity by downregulating insulin-like peptide expression and decreasing phosphorylation of downstream effectors, thereby increasing stress resistance and prolonging lifespan [7].

Q also reduces reactive oxygen species (ROS) production and upregulates genes involved in aging and ROS metabolism [7,9]. Given the high degree of homology between the IIS pathway in *C. elegans* and humans, including genes encoding transcription factors related to aging and senescence [10,11,12,13,14,15], the data obtained from this preclinical model are highly informative. Due to its characteristics, such as simplicity, transparency, ease of cultivation, short lifespan, and conserved, metabolically active digestive system, this nematode serves as a unique tool for quickly obtaining relevant information on the physiological and pathological mechanisms underlying aging and changes in stress resistance, and for evaluating the biological and molecular protective effects of various diet-derived compounds [11,16].

Recently, Q has been proposed as a natural senolytic compound that reduces the number of senescent cells in vitro and in vivo in mice [17,18,19,20]. However, its senolytic potential is weaker than that of other senolytic agents [19]. This was attributed to specific characteristics of Q, such as its low stability, which varies with temperature, pH, metal ion concentrations, and glutathione level. In addition, Q is difficult to absorb into the body and has a rapid metabolism, making it hard to achieve the high tissue levels necessary for senolytic activity in vivo. Additionally, its poor water solubility complicates its oral bioavailability and may limit the benefits of this flavonoid, thereby restricting its practical use.

Several attempts have been made to improve Q bioavailability, including the use of liposomes, nanoparticles, nanoemulsions, and micelles [21,22]. Among the various delivery systems, a lecithin carrier-based (Phytosome™) formulation of 100% natural Q [23] called Quercefit™ (QF) was developed [24]. This phytosome delivery system was designed to naturally enhance the solubility of poorly bioavailable natural active ingredients and their ability to cross biological barriers, thereby improving bioavailability and, consequently, biological activity. When administered to healthy volunteers, QF enables Q absorption up to 20 times that of the unformulated version, without any noticeable side effects [24].

For the first time, our study tested the hypothesis that the QF can enhance the potential anti-aging effect in vivo using the nematode *C. elegans*. Experiments were conducted under both physiological and stress-induced conditions to compare the impact of QF versus an unformulated Q extract on slowing the organism’s natural aging and protecting it from the harmful effects of stressors. We also assessed QF’s ability to reduce the accumulation of advanced glycation end products (AGEs), which are considered markers of senescence [25,26,27], and to modify the expression of genes encoding components of the IIS pathway. We analyzed the effect of QF on the ortholog of the human forkhead box O4 (FOXO4) transcription factor, *daf-16*, which is a key mediator of longevity because its activation can result in lifespan extension [8]. Its target genes mediate the oxidative and heat shock stress responses; these include superoxide dismutase-3 (*sod-3*), catalase-1 (*ctl-1*), and small heat shock proteins (*hsp*)*-16.2* and *hsp-70* [7,9]. In addition, transcription factors relevant to the regulation of stress-responsive genes involved in longevity and immunosenescence, namely, *skn-1* (an ortholog of the mammalian Nrf/CNC proteins), which encodes detoxification enzymes that scavenge ROS, and *hsf-1* (an ortholog of the human heat shock transcription factor 1), were considered [28].

The findings support the notion that QF, more than unformulated Q, can promote stress resistance and longevity in *C. elegans* under stressful conditions by modulating gene expression and heat-shock-element activity.

## 2. Results

### 2.1. Optimization of Experimental Conditions for QF’s Efficacy Studies

**Figure 1:**
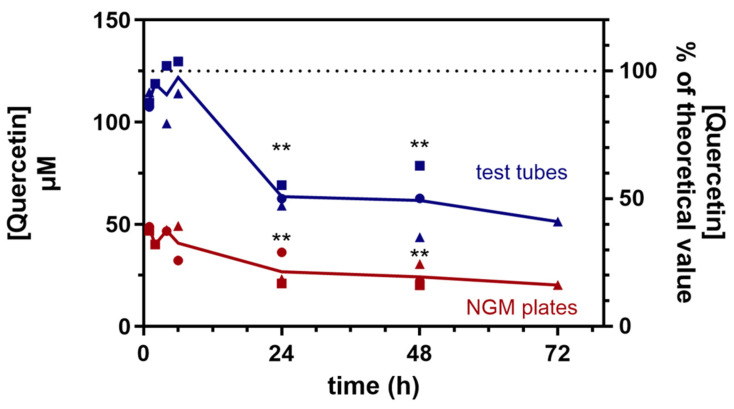
Stability of Q in QF. The stability of Q in QF was measured at different time points after incubation in NGM plates, simulating the conditions used in studies with the worms, or in test tubes for comparison. Three independent experimental sessions were carried out with ex-novo-prepared solutions, and the results of each session are indicated by a different shape: circle for the 1st session, square for the 2nd session, and triangle for the 3rd session. Dotted line: theoretical value at t = 0. Each of these points is the mean of three intra-day replications; SD is not reported for clarity but it was always < 25%. ** *p* < 0.01 vs. t = 1 h (one-way ANOVA, carried out only for groups with three values).

Initial studies were conducted to establish the stability of Q under the experimental conditions required to assess the effects of QF in *C. elegans*. To achieve this, Q and QF were dissolved in 1% carboxymethyl cellulose (CMC) and 0.3% polysorbate-80 to make a 2 mM Q solution, which was then diluted fourfold in OP50 *E. coli* and spotted onto Nematode Growth Medium (NGM) agar plates (250 µL/plate) or aliquoted into test tubes (250 µL/tube) for comparison. After incubation for 1 to 72 h, the plates were washed with 1 mL of M9, and the samples in the tubes were diluted to 1 mL with M9. Figure 1 shows that the concentration of Q in QF measured in the tubes remained stable for up to 6 h, remaining close to the expected value of 125 µM (90–97% on average). The concentration dropped to approximately 50% after 24 h, and then remained mostly stable until 72 h. In samples recovered by washing NGM plates, the Q concentration remained nearly constant for the first 6 h; however, it accounted for only 33–38% of the amount of QF initially applied to the plates, possibly due to bacterial uptake and/or incomplete recovery during washing.

We also observed that approximately 50% of Q in QF degraded between 6 and 24 h (as seen in tubes), with no further degradation until 72 h. Under these conditions, unformulated Q remained stable for up to 48 h of incubation, with a small drop occurring at 72 h (101 ± 6% and 73 ± 3% of t = 0, respectively; *N* = 3 replicates). This difference could be ascribed to the fact that the QF formulation can be taken up and degraded by bacteria more readily than the unformulated Q.

Based on these results and taking into account that practical reasons prevented us from avoiding the partial degradation occurring in the first 24 h, for the efficacy studies, we planned to keep the worms on NGM plates containing equimolar concentrations of unformulated and QF-formulated Q for a maximum of 48 h, after which, they were transferred to new, fresh plates prepared in the same manner.

Based on these results, experiments were conducted by dissolving unformulated Q and QF in 1% CMC containing 0.3% polysorbate-80 (Vehicle) to obtain a stock solution containing 2 mM Q, which was then diluted to 50–200 µM in live OP50 *E. coli* before being seeded on NGM plates. Synchronized worms were cultured on these plates and transferred every 48 h to new plates containing fresh OP50 *E. coli* with the test products, ensuring continuous exposure to a stable concentration of either formulated or non-formulated phytonutrients. Control worms were cultured on NGM plates seeded with OP50 *E. coli*, which contained the same volume of vehicle alone.

### 2.2. QF Enhances Worm Resistance to Thermal and Oxidative Stress

**Figure 2:**
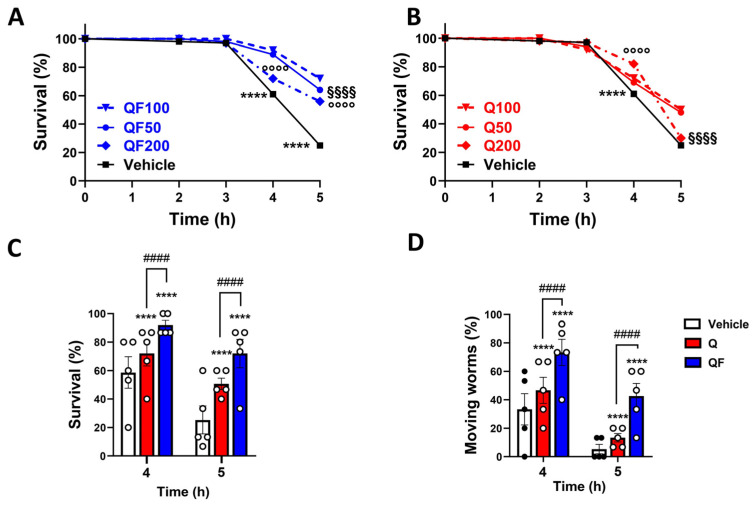
QF is more effective than unformulated Q in increasing heat stress tolerance. Synchronized worms were grown for 72 h at 20 °C on NGM agar plates seeded with (**A**) QF (blue) or (**B**) equimolar Q (red) dissolved in 1% CMC and 0.3% polysorbate 80 and diluted with OP50 *E. coli* to administer 0, 100, or 200 µM Q. Control worms were maintained on NGM plates seeded with OP50 *E. coli* and 1% CMC and 0.3% polysorbate 80 alone (Vehicle). Thermal stress was induced by incubating worms at 35 °C, and survival and motility were evaluated at different time points thereafter. (**A**,**B**) Percentage of survival expressed as percentage of viable worms at time 0. Data are the mean ± SEM from 5 independent experiments (*N* = 75). **** *p* < 0.0001 for vehicle vs. all concentrations of treated worms at the corresponding time point, °°°° *p* < 0.0001 for 200 µM vs. 50 and 100 µM QF or Q-treated worms at the corresponding time point, and (**A**) §§§§ *p* < 0.0001 for 50 µM QF vs. 100 and 200 µM QF and (**B**) §§§§ for *p* < 0.0001 200 µM Q vs. 50 and 100 µM Q at the corresponding time point (one-way ANOVA and Bonferroni post hoc analysis). (**C**) Percentages of surviving and (**D**) moving worms determined after 4 and 5 h of thermal stress in untreated worms (Vehicle) or worms treated with 100 µM unformulated Q or QF. Data are the mean ± SEM from 5 independent experiments (*N* = 75). **** *p* < 0.0001 vs. Vehicle at the corresponding time point, and #### *p* < 0.0001 (one-way ANOVA and Bonferroni post hoc analysis).

The effect of QF on worms’ ability to resist acute thermal and oxidative stress (immediate impacts on viability and motility) was first evaluated. Specifically, nematodes grown in the presence of 50–200 µM QF or equimolar Q were subjected to thermal stress on the third day of adulthood, and their survival was assessed at various time points thereafter. The average proportion of living worms remained unchanged until three hours after the stress across all groups (Figure 2A,B). Exposure to thermal stress for 4 and 5 h resulted in a significant reduction in worm survival in the control group, decreasing to 61% and 25%, respectively, while worms treated with all doses of QF or unformulated Q exhibited significantly higher survival.

Although a concentration-dependent protective effect was observed at 50 and 100 µM, the highest dose, 200 µM, showed a weaker effect. Five hours after the stress, only 56% of worms treated with 200 µM QF remained alive, compared with 64% and 72% for 50 and 100 µM, respectively (Figure 2A). A similar trend was also observed when worms were administered unformulated Q [7,29] (Figure 2B). The reduced protective effect observed at the highest dose of Q and QF may reflect a hormetic response, characterized by a biphasic dose–response relationship in which low or moderate doses of a compound induce beneficial adaptive responses. In contrast, higher doses may result in toxic effects [30]. We observed that the hormetic response was less evident when Q was administered in the QF formulation. Based on these data, we selected 100 µM as the optimal dose of QF and unformulated Q for all subsequent experiments. Interestingly, at this dose level, QF was significantly more effective than the same dose of unformulated Q in protecting worms from death (Figure 2C) and movement impairment (Figure 2D) induced by thermal stress.

**Figure 3:**
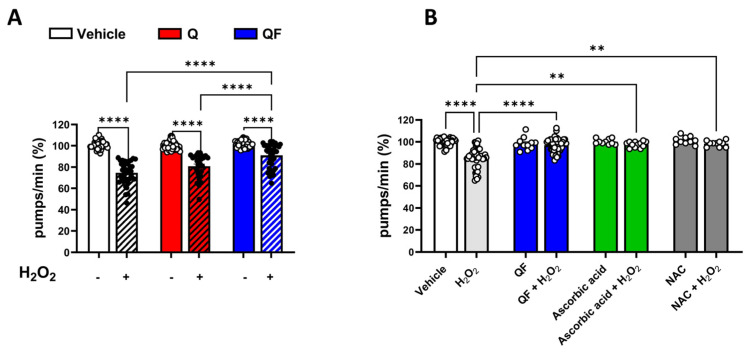
QF increased the resistance to oxidative stress. Synchronized worms were grown for 72 h at 20 °C on NGM agar plates seeded with QF or unformulated Q dissolved in 1% CMC containing 0.3% polysorbate 80 (Vehicle) and diluted with OP50 *E. coli* to obtain 100 µM Q. Control worms were maintained on NGM plates seeded with OP50 *E. coli* and the corresponding volume of vehicle. Oxidative stress was induced by treating worms with 0.5 mM H_2_O_2_ for 2 h at 20 °C, and (**A**) pharyngeal motility was evaluated 24 h later. Pharyngeal pumping of worms is expressed as the percentage of pumps/min of vehicle-treated worms. Data are the mean ± SEM from 4 independent experiments (*N* = 40). (**B**) To evaluate the protective effect of antioxidant compounds, worms were treated for 2 h at 20 °C with 0.5 mM hydrogen peroxide in the absence or presence of 284 µM ascorbic acid and 5 mM N-acetylcysteine (NAC), and pharyngeal motility was determined 24 h later. Pharyngeal pumping of worms is expressed as a percentage of pumps/min of vehicle-treated worms. Data are the mean ± SEM from 3 independent experiments (*N* = 30). ** *p* < 0.005, and **** *p* < 0.0001 (one-way ANOVA and Bonferroni post hoc analysis).

Muscle dysfunction, including decreased motility and feeding behavior, is considered a marker of aging and senescence and is known to worsen under stress conditions, such as heat exposure and ROS stress [31,32,33,34]. Worms grown in the presence of QF were treated with hydrogen peroxide on the first day of adulthood, and the function of the pharyngeal muscles was assessed 24 h later. At 100 µM, QF but not Q significantly protected worms from the pharyngeal dysfunction caused by 0.5 mM hydrogen peroxide (Figure 3A). This effect is similar to that observed when worms were administered prototypic antioxidants, such as 5 mM N-acetylcysteine (NAC) and 284 µM ascorbic acid (Figure 3B). QF and unformulated Q, as well as NAC and ascorbic acid, at the concentrations used, did not affect the feeding behavior of the worms (Figure 3A,B).

### 2.3. QF Enhances the Worm’s Lifespan and Healthspan

**Figure 4:**
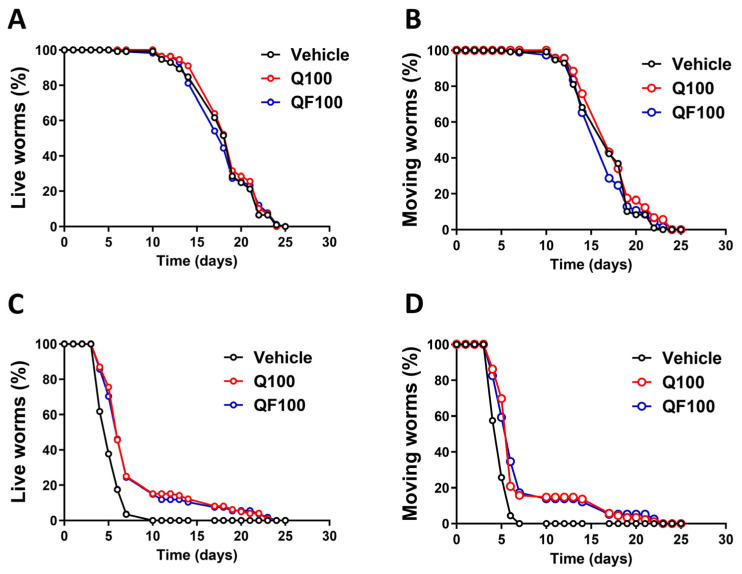
Effect of QF and unformulated Q on the lifespan and healthspan of *C. elegans* under physiological and heat stress damage conditions. (**A**,**B**) Synchronized worms (40 worms/group) were plated on NGM plates seeded with OP50 *E. coli* and 100 µM Q or an equimolar concentration of QF dissolved in a solution containing 1% CMC and 0.3% polysorbate 80 and left at 20 °C. Control worms were plated on NGM plates seeded with OP50 *E. coli* and 1% CMC and 0.3% polysorbate 80 (Vehicle). The (**A**) lifespan and (**B**) healthspan were scored daily. (**C**,**D**) Synchronized worms (40 worms/group) were grown for 72 h at 20 °C on NGM agar plates seeded with 100 µM Q or an equimolar concentration of QF dissolved in 1% CMC and 0.3% polysorbate 80 and diluted with OP50 *E. coli*. Control worms were maintained on NGM plates seeded with OP50 *E. coli*, 1% CMC, and 0.3% polysorbate 80 alone (Vehicle). Thermal stress was induced by incubating worms at 35 °C for 3 h. Worms were then transferred to 20 °C on NGM agar plates seeded with 100 µM Q, an equimolar concentration of QF, or Vehicle. The (**C**) lifespan and (**D**) healthspan were scored daily. Data are the mean ± SEM. See Table 1 for mean lifespan, healthspan, and statistical analyses.

The effect of QF on the lifespan and healthspan of worms was evaluated under physiological conditions. In addition, its effect under post-stressed conditions was evaluated to assess the long-term consequences of thermal stress. In the presence or absence of 100 µM QF or unformulated Q, no extension in lifespan or improvement in healthspan was observed under physiological conditions (Figure 4A,B and Table 1). However, under thermal stress conditions, 100 µM QF, similar to unformulated Q, increased the worms’ lifespan and healthspan by about 50%, demonstrating its capacity to counteract the decline in lifespan and healthspan in response to stress (Figure 4C,D and Table 1). Since a decrease in worm motility is a functional parameter indicative of aging, the ability of QF and unformulated Q to extend healthspan suggests a senolytic effect.

### 2.4. QF Does Not Significantly Reduce AGE Accumulation

**Figure 5:**
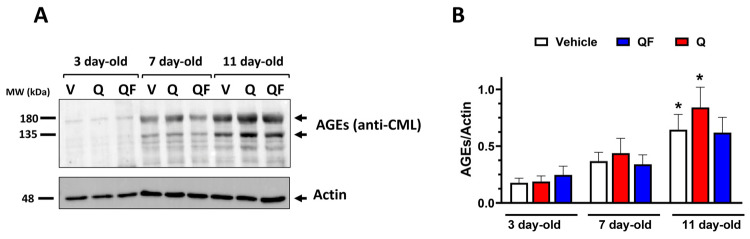
Effect of QF and Q on AGEs. (**A**) Western Blot, representative of 3 biological replicates, of AGEs in samples extracted from worms of different ages grown at 20 °C in the presence of 100 µM QF or Q dissolved in a solution containing 1% CMC and 0.3% polysorbate 80. Control worms were grown under the same experimental conditions in the presence of 1% CMC and 0.3% polysorbate 80 (Vehicle, V). Proteins extracted from 20 worms were loaded in each gel lane and immunoblotted with anti-AGE-CML or anti-actin antibody. (**B**) Quantification of total AGEs is expressed as the mean volume of anti-AGE-CML band (at 135 and 180 kDa) immunoreactivity normalized to that of the actin band. Data are mean ± SEM (*N* = 5), * *p* < 0.005 vs. the corresponding group at 3 days of adulthood (two-way ANOVA and Bonferroni post hoc analysis).

The senolytic effects of Q and QF were assessed by measuring AGE accumulation, a marker of senescence. As expected, we observed a significant increase in AGEs with increasing worm age starting on the 11th day of adulthood, as quantified by Western Blot analysis using the anti-AGE CML antibody (Figure 5A,B). Neither QF nor Q significantly reduced AGE accumulation. However, in 11-day-old worms grown with 100 µM Q, a significant accumulation of AGEs was observed, similar to that in the controls, whereas in the presence of QF, no such accumulation was observed (Figure 5B). The lack of significance for QF could be due to high variability in the Western Blot data, caused by the poor specificity of the anti-AGE antibody, or it may reflect a more significant effect emerging later as the worms age.

### 2.5. QF Under Stress Conditions Regulates Gene Expression and Heat-Shock Elements

**Figure 6:**
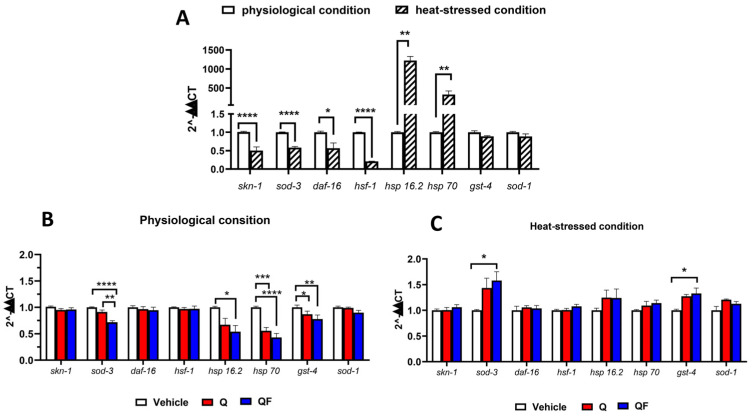
QF modulates the expression of genes associated with longevity and stress resistance. (**A**) Expression level of the analyzed genes in worms exposed to thermal stress (heat-stressed conditions) or physiological conditions. Data are expressed as the mean ± SEM, normalized to the value obtained under physiological conditions. Data are from 3 independent experiments (*N* = 15). * *p* < 0.005, ** *p* < 0.001, and **** *p* < 0.0001 (two-way ANOVA and Bonferroni post hoc analysis). (**B**,**C**) Effect of Q and QF on the expression of genes in worms (**B**) maintained under physiological conditions and (**C**) exposed to thermal stress. Data are expressed as the mean ± SEM from 3 independent experiments (*N* = 15), and are normalized to the value obtained with vehicle. * *p* < 0.005, ** *p* < 0.001, *** *p* < 0.0005, **** *p* < 0.0001 (two-way ANOVA and Bonferroni post hoc analysis).

To understand the mechanism of QF activity, we measured gene expression in the IIS and MAPK pathways using RT-qPCR in worms exposed to thermal stress. Heat exposure significantly increased the expression of *hsp-16.2* and *hsp-70*, while decreasing the transcript levels of *skn-1* (Figure 6A) [35]. We also observed a decrease in *daf-16, hsf-1*, and *sod-3* (Figure 6A), which are stress-response genes typically upregulated by thermal stress. Since the transcriptional response to heat stress can vary depending on experimental conditions, including the duration and intensity of heat exposure, the developmental stage of the worms, and the timing of RNA collection after stress [36], we cannot exclude the possibility that an increase in their protein levels could mediate downregulation *daf-16*, *hsf-1*, and *sod-1* gene expression.

As shown in Figure 6B,C, QF, like Q, did not change the expression of *skn-1*, *daf-16*, *hsf-1*, and *sod-1* in worms under normal or heat-stressed conditions. In worms maintained under physiological conditions, QF significantly reduced the expression of the *sod-3*, *hsp-16.2*, *hsp-70*, and *gst-4* genes compared to control nematodes. This effect may reflect reduced activation of stress-response pathways, possibly due to its antioxidant properties, although other mechanisms cannot be excluded. Although to a lesser extent, similar effects were observed in worms treated with an equimolar concentration of Q; however, for *hsp-16.2*, the decrease did not reach statistical significance (Figure 6B).

As suggested for Q [37], QF may also act as a ROS scavenger, thus repressing the transcription of heat-shock element genes activated by thermal stress, such as *hsp-16.2* and *hsp-70* (Figure 6A), as well as genes involved in the antioxidant and detoxification response, like *sod-3* and *gst-4*. Notably, in worms subjected to thermal stress, QF significantly promoted the expression of these last two genes and slightly increased, although not significantly, the expression of *hsp-16.2* (Figure 6C). As reported in the literature, less pronounced effects that did not reach statistical significance were observed with Q (Figure 6C) [7]. These results indicate that, under stressed conditions, QF can modulate gene levels and heat-shock elements associated with longevity and stress resistance.

## 3. Discussion

Flavonoids are proposed to delay and palliate aging where senescence is involved. The natural phenolic compounds from dietary sources that protect against the aging process include, among others, naringenin, hesperidin, Q, kaempferol, luteolin, genistein, epigallocatechin gallate, and resveratrol. Many of these compounds possess anti-senescence effects. The benefits of Q in combating aging, including protection against oxidative stress-induced cellular damage, are well documented. However, they may be limited in vivo by Q’s low water solubility, poor stability, and poor oral bioavailability, requiring a high dose to achieve desired effects. A lecithin formulation technology has been used to develop QF to improve the water solubility of Q and enhance its in vivo biological absorption. We confirmed here, using *C. elegans*, that this formulation improves the anti-aging effect of Q. Since the empty lecithin-based formulation alone was not administered to worms, it cannot be excluded that other components in the phytosome delivery system, in addition to Q itself, may contribute to the beneficial effects of QF.

A comparison of the QF effects with other Q formulations is difficult because of the lack of data on stress resistance and longevity in healthy wild-type worms. The only study available investigated the effect of a nanosized food-grade Q-loaded nanoemulsion system on the transgenic *C. elegans* strain NL5901, a model of Parkinson’s disease [38]. In this paper, the authors show that the formulation reduced α-synuclein aggregation, increased mitochondrial and fat contents, and improved lifespan [38].

We observed that QF, despite the partial degradation observed under our experimental conditions, still exerted a greater beneficial effect than Q in most assays. Indeed, QF is more effective than Q in protecting worms against thermal and oxidative stress-induced toxicity, with effects similar to those of common antioxidants such as ascorbic acid and NAC. QF also significantly extends the lifespan and healthspan of stressed worms, but not those of nematodes aged under physiological conditions. These results do not align with the available literature, which reports a slight but significant increase in the mean lifespan of approximately 10% when worms were grown in the presence of a comparable concentration of Q [8,9]. However, it should be noted that the published data were obtained using fluorodeoxyuridine to render worms sterile. This carcinogenic compound, whose use is debated among *C. elegans* researchers, could act as a stressor; therefore, the results obtained under these conditions may reflect stress-induced rather than physiological aging.

Gene expression analyses also indicate that the effects of QF are linked to increased expression of *sod-3* and *gst-4*, which are associated with longevity and stress resistance, and to a slight, although non-significant, increase in *hsp-16.2* expression, which is also associated with longevity. These results align with data from other groups studying the effect of Q in wild-type and mutant worms [7,8,37,39,40], but differ from the findings of Sugawara and Sakamoto, who reported that Q feeding increased the transcription of all these genes [9]. The reason for this difference is difficult to determine and likely relates to differences in methods and experimental approaches. Notably, although *skn-1* is a key gene for regulating detoxification enzymes and is reported to be involved in Q-induced resistance to thermal stress [9], its expression level was unaffected by QF, especially in this model [7,39]. Additionally, QF did not alter the expression of *sod-1*, a *skn-1* target gene encoding ROS scavengers, which was only reported to be increased by Q by Sugawara and Sakamoto [9]. The protective effect of QF was not associated with changes in *daf-16* gene expression [7,8]. Still, it cannot be excluded that, as suggested for Q, it is related to the protein’s nuclear translocation, which then activates the expression of stress-responsive genes [37].

Bacteria, used as food sources for *C. elegans*, provide essential nutrients but can also activate symbiotic interactions, which are important factors in studies on aging and longevity [41]. To avoid this problem, inactivation of bacteria by different methods, i.e., heat, UV, paraformaldehyde, and antibiotics, has been proposed. However, none of these treatments is without limitations [42]. For example, heat inactivation reduces the palatability of *E. coli OP50*, altering normal food ingestion and potentially affecting the physiological development of worms, as well as activating the mitochondrial unfolded protein response, decreasing lipid stores, and reducing fertility [42]. Paraformaldehyde-inactivated bacteria affect brood size, development, and food preference, and inactivation by UV and antibiotics affects lifespan and other relevant phenotypes in worms [42]. This was the reason we decided to feed the worms live OP50 *E. coli* and apply the same experimental conditions across all the experimental groups in the study to minimize the possible contribution of bacteria to the results on the effect of Q and QF.

In conclusion, this is the first evidence that QF in *C. elegans*, acting at the transcriptional level, can provide stress protection that slows aging. Clinical studies are planned to explore and translate the promising lifespan benefits observed in our research into human applications.

## 4. Materials and Methods

### 4.1. Materials

Methanol, acetonitrile (ACN), formic acid, phosphoric acid, and polysorbate-80 (cod. P1754) were obtained from Sigma-Aldrich Co. (Milan, Italy). Sodium carboxymethyl cellulose (CMC, cod. 14094) was obtained from Farmalabor (Assago, Milan, Italy). Potassium dihydrogen phosphate, sodium phosphate dibasic, sodium chloride, and magnesium sulphate were obtained from Merck (Rahway, NJ, USA). A Milli-Q system was used to produce HPLC-grade water in-house (Millipore, Bedford, MA, USA). Food-grade quercetin from the flower of *Sophora japonica* L. (100%) (Q) and Quercefit™ (a Quercetin Phytosome™) (QF) were provided by Indena S.p.A (Milan, Italy). QF was prepared using Indena Phytosome™, a proprietary, multi-talented Good Manufacturing Process technology platform designed to enhance the bioavailability of phytonutrients. QF formulation consists of ~40% Q, ~40% sunflower lecithin, 18% potato maltodextrin, and 2% silicon dioxide (Patent Application No. WO2019/016146). According to the manufacturer’s datasheet, the particle size dimensions for QF were D10: 21 µm, D50: 140 µm, D90: 496 µm. During the manufacturing process, no new covalent bonds formed between the constituents, and the composition or structure of Q remained unchanged. QF standardization is 34.0–42.0% of Q according to HPLC.

### 4.2. Quercetin Stability

Q is usually administered to worms by mixing it with OP50 *Escherichia coli* (Caenorhabditis Genetics Centre (CGC), University of Minnesota, Minneapolis, MN, USA). To establish optimal experimental conditions ensuring equimolar administration of unformulated and formulated Q to *C. elegans*, a stability analysis of Q in the lecithin-based formulation, as well as unformulated Q, was conducted. QF and Q were dissolved in 1% CMC containing 0.3% polysorbate-80 to obtain a solution containing 2 mM Q. The solutions were diluted to 500 µM Q (150 µg/mL) in OP50 *E. coli* and spotted onto Nematode Growth Medium (NGM) agar plates (250 µL/plate). After drying, the plates were incubated at 20 °C for various time points ranging from one hour (i.e., the time required to dry the *E. coli* on the agar) to 2, 4, 6, 24, 48, and 72 h. The plates were then washed with 1 mL M9 buffer (0.3% potassium dihydrogen phosphate, 0.6% sodium phosphate dibasic, 0.5% sodium chloride, and 1 M magnesium sulfate), and the wash was collected for Q measurement. For comparison, 250 µL of QF solution containing 500 µM Q in OP50 *E. coli* was also incubated in test tubes at 20 °C for the same time points (1, 2, 4, 6, 24, 48, and 72 h), and then dispensed into tubes containing 750 µL of M9 to achieve a final volume of 1 mL. Each sample was prepared in triplicate. At the end of the incubations, all solutions were sonicated for 15 min at high intensity using a Bioruptor^®^ (Diagenode, SA, Seraing, Ougrée, Belgium), and then centrifuged at 1200× *g* for 10 min to remove cellular debris. The supernatants were collected and analyzed by HPLC with UV detection to quantify Q content, as previously described [24]. Unformulated Q was used for the calibration curve. To this end, Q was dissolved in methanol at a concentration of 1 mg/mL and serially diluted with methanol to obtain eight working solutions ranging from 1.0 to 200 µg/mL. Stock and working solutions of Q were stored at 4 °C. Chromatographic separation was performed on an X Bridge BEH C18 (150 × 4.6 mm, 2.5 µm) (Waters SpA, Sesto San Giovanni, Italy) equipped with an X Bridge BEH safeguard column (3.9 × 5 mm, 2.5 µm, Waters) maintained at 35 °C. The mobile phase consisted of 0.3% phosphoric acid in water and 100% ACN with a constant flow of 0.7 mL/min. Elution began with 75% mobile phase A (0.3% phosphoric acid in water) and 25% mobile phase B (100% ACN), followed by an 8 min linear gradient to 40% A, a 1 min linear gradient to 10% A, and a 1 min linear gradient back to 75% A, which was maintained for 5 min to equilibrate the column. The total run time was 15 min. The injection volume was 15 µL, and the wavelength was set at 300 nm for quantitative analysis. The retention time for Q was 6.8 min. Supplementary Figure S1 shows a representative chromatogram. The peak area was plotted against the corresponding Q concentration, and the linearity of a representative concentration-response fitting was obtained using a weighted 1/x^2^ linear function. A new calibration curve sample was prepared for each experimental session, consistently yielding an R^2^ > 0.99 and reproducible results. No UV signals were observed when injecting blank solutions containing M9 ± OP50 *E. coli* alone.

### 4.3. C. elegans Maintenance and Treatment

N2 Bristol *C. elegans* (Caenorhabditis Genetics Centre (CGC), University of Minnesota, Minneapolis, MN, USA) were cultured at 20 °C on NGM plates spread with live or inactivated OP50 *E. coli* (CGC) as food. QF and unformulated Q were dissolved in 1% CMC containing 0.3% polysorbate-80 to obtain a solution containing 2 mM Q. The solutions were diluted in OP50 *E. coli* and spotted onto NGM agar plates (250 µL/plate). Worms were synchronized by egg-laying and cultured at 20 °C on NGM plates spread with OP50 *E. coli* containing 50–200 µM Q in QF or the same concentration of unformulated Q. Control worms were fed OP50 *E. coli* containing the same volume of 1% CMC and 0.3% polysorbate-80 alone (Vehicle).

### 4.4. Lifespan and Healthspan

To evaluate the effect of the test compounds on the physiological decline in lifespan and healthspan, nematodes were synchronized by egg-laying and transferred to NGM plates seeded with *E. coli* in the presence or absence of QF and unformulated Q (equimolar concentration) daily during the fertile period (5–6 days) to avoid overlapping generations. During this initial period, the test compounds were added to NGM plates daily. For the subsequent period (approximately 15 days), they were added every 48 h based on the results of the stability studies. The effects on lifespan and healthspan under detrimental stress conditions were investigated in worms synchronized by egg-laying and transferred to NGM plates seeded with *E. coli*, either in the presence or absence of QF or the same concentration of unformulated Q (equimolar concentration, 100 µM Q) dissolved as described above. The worms were grown at 20 °C for 72 h. Control worms were only fed the vehicle. The nematodes were incubated at 35 °C for 3 h and then placed on 20 °C NGM plates seeded with fresh *E. coli*, again in the presence or absence of the tested product. The worms were transferred daily as described. Dead, alive, and censored animals were recorded during transfers. Animals were counted as dead if they neither moved nor reacted to manual stimulus with a platinum wire, and had no pharyngeal pumping activity. Animals with a ruptured vulva phenotype or those desiccated against the wall were censored. To determine the mean lifespan and survival curve, the counts of dead and censored animals were used for survival analysis using the Online Application for Survival Analysis (OASIS 2) [43]. The Kaplan–Meier estimator was employed, and *p*-values were calculated using the log-rank test to compare experimental groups. The number of active movements was also assessed in the nematodes used in the lifespan assay to assess health status. Animals crawling spontaneously or in response to manual stimulus were considered moving, while dead animals and those without crawling behavior were considered not moving. The statistical analysis was performed as described for lifespan [44].

### 4.5. Stress Assays

Synchronized worms were cultured for 72 h at 20 °C on NGM plates seeded with OP50 *E. coli*, either in the presence or absence of the test compound. Heat stress was induced by subjecting the animals to 35 °C for 1 to 5 h, while control worms were maintained at 20 °C (15 worms/group, 5 independent experiments). The initiation of heat stress was designated as time 0 h. Dead, alive, and censored animals were recorded at different time points. Animals were counted as dead if they neither moved nor reacted to manual stimulus with a platinum wire, and had no pharyngeal pumping activity. Animals with a ruptured vulva phenotype or those desiccated against the wall were censored. The survival rate was calculated using the Online Application for Survival Analysis OASIS 2 [43]. In addition, the number of active movements was also assessed. Animals crawling spontaneously or in response to manual stimulus were considered moving, while dead animals and those without crawling behavior were considered not moving. The statistical analysis was performed as described for the survival analysis. Oxidative stress was induced by collecting worms and treating them with 0.5 mM hydrogen peroxide (100 worms/100 µL) for 2 h at 20 °C, and plating them on fresh NGM plates seeded with OP50 *E. coli*, either in the presence or absence of the test compound. The feeding behavior of the worms was assessed 24 h later by scoring the pharyngeal pumping rate, counting the number of times the terminal bulb of the pharynx contracted in one minute (pumps/min) (10 worms/group, 4 independent experiments) [45].

### 4.6. AGEs Formation

Synchronized worms were cultured at 20 °C on NGM plates seeded with OP50 *E. coli*, either with or without the test compound. The formation of AGEs was evaluated as described by Komura et al., 2021 [46]. Briefly, 20 worms aged 3 to 11 days of adulthood were collected by picking, placed in M9 buffer, allowed to settle by gravity, and washed in M9 to remove bacteria. Worm pellets were suspended in 30 µL of 60 mM Tris solution, pH 6.8, containing 10% glycerol, 2% sodium dodecyl sulfate (SDS), and 5% β-mercaptoethanol; boiled for 10 min at 95 °C; and loaded into a 10% SDS-PAGE gel. Proteins were separated at 100 V in SDS running buffer and transferred for 2 h at 100 V onto a polyvinylidene fluoride membrane in 20 mM Tris solution containing 150 mM glycine and 10% methanol. Membranes were blocked for 1 h at room temperature in a 10 mM Tris-HCl solution, pH 7.5, containing 100 mM NaCl, 0.1% (*v*/*v*) Tween 20, 5% (*w*/*v*) low-fat dry milk powder, and 2% (*w*/*v*) bovine serum albumin, and incubated overnight at 4 °C with a mouse monoclonal anti-AGE antibody clone 6D12 (1:1000 dilution, DBA, Milan, Italy, catalog number KAL-KH001) or mouse monoclonal anti-actin antibody clone C4 (1:2000 dilution, Sigma-Aldrich, catalog number MAB1501). Anti-mouse IgG peroxidase conjugate (1:10,000, Sigma-Aldrich, catalog number A4416) was used as the secondary antibody. Chemioluminescence was detected using Clarity Max Western ECL Substrate (Biorad, Hercules, CA, USA), and the membranes were scanned with a ChemiDoc Imaging System (Biorad). The mean volumes of the immunoreactive bands were determined using Image Lab™ 6.1 software (Bio-Rad Laboratories, Hercules, CA, USA).

### 4.7. Gene Expression

Synchronized worms were cultured for 72 h at 20 °C on NGM plates seeded with OP50 *E. coli* in the presence or absence of the test compound and subjected to heat stress by incubating them at 35 °C for 3 h. Control worms were maintained at 20 °C. The nematodes were collected in M9 buffer, settled by gravity, and washed with M9 buffer to remove bacteria. RNA was extracted from the worm pellet using the Maxwell^®^ RSC simplyRNA Tissue kit (Promega Italia Srl, Milan, Italy) according to the manufacturer’s instructions. Briefly, the worm pellet was homogenized with a Turrax (T10, IKA-Werke GmbH & Co., Staufen im Breisgau, Germany) for 2 min using 200 µL of a chilled working solution prepared by adding 20 µL of 1-thioglycerol per milliliter of the homogenization solution. Lysis buffer (200 µL) was added to 200 µL of homogenate, vortexed for 15 s, and transferred into the cartridge well. RNA was extracted and eluted in 50 µL of nuclease-free water, and the concentration was quantified using a NanoDrop (Thermo Fisher Scientific Inc., Monza, Italy). mRNA was reverse transcribed using a High-Capacity cDNA Reverse Transcription kit (Applied Biosystems, Thermo Fisher Scientific). For this, 1 µg of RNA was retrotranscribed using random primers in a 20 µL final mix volume. cDNA (50 ng) was used for Q-PCR amplification using the 2X qPCR Master Mix Power SYBR^TM^ Green PCR (Applied Biosystem) and the Applied Biosystem QuantStudio^TM^ 5 Real-Time PCR System. Specific primers were designed from the gene sequences of interest and used for PCR amplification (Table 2). The relative gene expression levels were determined using the 2^−ΔΔCT^ method, with the cell division cycle-related gene *cdc-42* or the conserved iron-binding-related *y45f10d.4* gene as the housekeeping gene.

### 4.8. Statistical Analysis

Synchronized worms obtained by egg-laying were randomly distributed onto NGM plates containing the different treatments to avoid allocation bias. For each experiment, worms were transferred to plates in comparable numbers across groups. All the evaluations were performed blinded to the treatment groups. The data were analyzed using GraphPad Prism 10.2 software (GraphPad, San Diego, CA, USA) and Student’s *t*-test, one-way or two-way ANOVA, and Bonferroni’s post hoc test. A *p*-value < 0.05 was considered significant. For lifespan and healthspan studies, the number of dead and censored animals was used for survival analysis in OASIS 2 [43]. The *p*-values were calculated using the log-rank and Bonferroni’s post hoc test on the pooled populations of animals.

## Figures and Tables

**Table 1:** QF and unformulated Q only affect worms’ survival and healthspan under heat stress damage conditions.

	PHYSIOLOGIC CONDITIONS			HEAT STRESS CONDITIONS		
	Vehicle	Q	QF	Vehicle	Q	QF
Sample size	120	120	120	120	123	120
Censored	11	16	18	3	8	17
Median of survival (days ± SEM)	18.28 ± 0.32	18.80 ± 0.29	18.21 ± 0.33	5.27 ± 0.13	8.16 ± 0.46 ****	7.94 ± 0.48 ****
Lifespan Improvement rate (%)	-	2.8	0	-	54.8	50.7
Median of moving Worms (days ± SEM)	16.76 ± 0.29	17.43 ± 0.30	16.57 ± 0.30	4.88 ± 0.08	7.45 ± 0.43 ****	7.49 ± 0.48 ****
Healthspan improvement rate (%)	-	4.0	0	0	52.7	53.5

Data are the mean ± SEM. **** *p* < 0.0001 vs. the corresponding vehicle according to log-rank and Bonferroni’s post hoc test between the pooled animal populations. The data were obtained from 40 worms/group, three independent experiments, with *N* = 120 for experiments under physiological conditions and *N* = 120 for experiments under heat stress. The background color distinguish the lifespan from healthspan data.

**Table 2:** List of the primers used in the study’s gene expression analysis.

Name	Sequence (5′-3′)
*skn-1*-forward	5′-GCGACGAGACGAGACGATAA-3′
*skn-1*-reverse	5′-TGAGGTGTTGGACGATGGTG-3′
*sod-3*-forward	5′-GGCTGTTTCGAAAGGGAATCT-3′
*sod-3*-reverse	5′-CCTTTGAAGGTTCTCCACCA-3′
*daf-16*-forward	5′-ATCAGACATCGTTTCCTTCGG-3′
*daf-16*-reverse	5′-TTAACCGTTTCTCTGGACTAGC-3′
*hsf-1*-forward	5′-CGAGGATCCACTCAGACAGC-3′
*hsf-1*-reverse	5′-GTAGTTTGGGTCCGGCACAT-3′
*hsp-16.2*-forward	5′-TCCATCTGAGTCTTCTGAGATTGTT-3′
*hsp-16.2* -reverse	5′-GATAGCGTACGACCATCCAAA-3′
*hsp-70*-forward	5′-AGCCGGTTGAAAAGGCACT-3′
*hsp-70*-reverse	5′-AGTTGAGGTCCTTCCCATTGAA-3′
*gst-4*-forward	5′-CTTGGCAAGAAAATTTGGACTC-3′
*gst-4*-reverse	5′-GCGTCACTTCCATAGAAAACG-3′
*sod-1*-forward	5′-AGGTCTCCAACGCGATTTTT-3′
*sod-1*-reverse	5′-TCGGACTTCTGTGTGATCCAG-3′
*cdc-42*-forward	5′-CTGTTGTGGTGGGTCGAGAG-3′
*cdc-42*-reverse	5′-GTTGACGCAGAAGGGACTGA-3′
*y45F10d.4*-forward	5′-ATCTTCCCTGGCAACCGAAT-3′
*y45F10d.4*-reverse	5′-TGGGCGAGCATTGAACAGT-3′

## Data Availability

The original contributions presented in this study are included in the article/Supplementary Materials. Further inquiries can be directed to the corresponding authors.

## Supplementary Materials

The following supporting information can be downloaded at: https://www.mdpi.com/article/10.3390/ph19040525/s1, Supplementary Figure S1: Representative chromatogram obtained with a solution of 1 µg/mL quercetin (i.e., the lowest point of the calibration curve).

## Abbreviations

The following abbreviations are used in this manuscript:

Q	Quercetin
QF	Quercefit™
ROS	Reactive oxygen species
*C.*	*Caenorhabditis*
IIS	Insulin/insulin-like growth factor signaling (IIS) pathway
AGEs	Advanced glycation end products
FOXO4	Human forkhead box O4
SOD-3	Superoxide dismutase-3
CTL-1	Catalase-1
HSP	Small heat shock protein
ACN	Acetonitrile
CMC	Sodium carboxymethyl cellulose
CGC	Caenorhabditis Genetics Centre
NGM	Nematode Growth Medium
SDS	Sodium dodecyl sulfate
NAC	N-acetylcysteine

## References

[1] Viña J., Olaso-Gonzalez G., Arc-Chagnaud C., De la Rosa A., Gomez-Cabrera M.C. (2020). Modulating Oxidant Levels to Promote Healthy Aging. Antioxid. Redox Signal..

[2] Wissler Gerdes E.O., Zhu Y., Tchkonia T., Kirkland J.L. (2020). Discovery, Development, and Future Application of Senolytics: Theories and Predictions. FEBS J..

[3] Ebrahimpour S., Zakeri M., Esmaeili A. (2020). Crosstalk between Obesity, Diabetes, and Alzheimer’s Disease: Introducing Quercetin as an Effective Triple Herbal Medicine. Ageing Res. Rev..

[4] McHugh D., Gil J. (2018). Senescence and Aging: Causes, Consequences, and Therapeutic Avenues. J. Cell Biol..

[5] Rothwell J.A., Perez-Jimenez J., Neveu V., Medina-Remón A., M’hiri N., García-Lobato P., Manach C., Knox C., Eisner R., Wishart D.S. (2013). Phenol-Explorer 3.0: A Major Update of the Phenol-Explorer Database to Incorporate Data on the Effects of Food Processing on Polyphenol Content. Database.

[6] Wang W., Sun C., Mao L., Ma P., Liu F., Yang J., Gao Y. (2016). The Biological Activities, Chemical Stability, Metabolism and Delivery Systems of Quercetin: A Review. Trends Food Sci. Technol..

[7] Ayuda-Durán B., González-Manzano S., Miranda-Vizuete A., Sánchez-Hernández E., Romero M.R., Dueñas M., Santos-Buelga C., González-Paramás A.M. (2019). Exploring Target Genes Involved in the Effect of Quercetin on the Response to Oxidative Stress in *Caenorhabditis elegans*. Antioxidants.

[8] Saul N., Pietsch K., Menzel R., Steinberg C.E.W. (2008). Quercetin-Mediated Longevity in *Caenorhabditis elegans*: Is DAF-16 Involved?. Mech. Ageing Dev..

[9] Sugawara T., Sakamoto K. (2020). Quercetin Enhances Motility in Aged and Heat-Stressed *Caenorhabditis elegans* Nematodes by Modulating Both HSF-1 Activity, and Insulin-like and P38-MAPK Signalling. PLoS ONE.

[10] Zhang S., Li F., Zhou T., Wang G., Li Z. (2020). *Caenorhabditis elegans* as a Useful Model for Studying Aging Mutations. Front. Endocrinol..

[11] Fabian D.K., Fuentealba M., Dönertaş H.M., Partridge L., Thornton J.M. (2021). Functional Conservation in Genes and Pathways Linking Ageing and Immunity. Immun. Ageing.

[12] Lan W., Xiao X., Nian J., Wang Z., Zhang X., Wu Y., Zhang D., Chen J., Bao W., Li C. (2024). Senolytics Enhance the Longevity of *Caenorhabditis elegans* by Altering Betaine Metabolism. J. Gerontol. Ser. A Biol. Sci. Med. Sci..

[13] Yarmey V.R., San-Miguel A. (2024). Biomarkers for Aging in *Caenorhabditis elegans* High Throughput Screening. Biochem. Soc. Trans..

[14] Wang H., Kern C.C., Nguyen Hong C., Saez Allende A., Qiao J., Zhang A., Fan Y., Ezcurra M., Gems D. (2026). A Hierarchy of Causes of Death in Senescent *C. elegans*. npj Aging.

[15] Mytilinaiou E., Kitopoulou K., Palikaras K., Gorgoulis V.G., Cavinato M., Evangelou K. (2025). *Caenorhabditis elegans* as a Screening Platform for Anti-Aging Compounds. Oncogene-Induced Senescence.

[16] Giunti S., Andersen N., Rayes D., De Rosa M.J. (2021). Drug Discovery: Insights from the Invertebrate *Caenorhabditis elegans*. Pharmacol. Res. Perspect..

[17] Hohmann M.S., Habiel D.M., Coelho A.L., Verri W.A., Hogaboam C.M. (2019). Quercetin Enhances Ligand-Induced Apoptosis in Senescent Idiopathic Pulmonary Fibrosis Fibroblasts and Reduces Lung Fibrosis In Vivo. Am. J. Respir. Cell Mol. Biol..

[18] Kim S.R., Jiang K., Ogrodnik M., Chen X., Zhu X.-Y., Lohmeier H., Ahmed L., Tang H., Tchkonia T., Hickson L.J. (2019). Increased Renal Cellular Senescence in Murine High-Fat Diet: Effect of the Senolytic Drug Quercetin. Transl. Res..

[19] Özsoy Gökbilen S., Becer E., Vatansever H.S. (2022). Senescence-Mediated Anticancer Effects of Quercetin. Nutr. Res..

[20] Watkins B.A., Mitchell A.E., Shin A.C., Dehghani F., Shen C.-L. (2025). Dietary Flavonoid Actions on Senescence, Aging, and Applications for Health. J. Nutr. Biochem..

[21] Tomou E.-M., Papakyriakopoulou P., Saitani E.-M., Valsami G., Pippa N., Skaltsa H. (2023). Recent Advances in Nanoformulations for Quercetin Delivery. Pharmaceutics.

[22] Ferreira-Silva M., Faria-Silva C., Carvalheiro M.C., Simões S., Marinho H.S., Marcelino P., Campos M.C., Metselaar J.M., Fernandes E., Baptista P.V. (2022). Quercetin Liposomal Nanoformulation for Ischemia and Reperfusion Injury Treatment. Pharmaceutics.

[23] Kara M., Sahin S., Rabbani F., Oztas E., Hasbal-Celikok G., Kanımdan E., Kocyigit A., Kanwal A., Wade U., Yakunina A. (2024). An In Vitro Analysis of an Innovative Standardized Phospholipid Carrier-Based *Melissa officinalis* L. Extract as a Potential Neuromodulator for Emotional Distress and Related Conditions. Front. Mol. Biosci..

[24] Riva A., Ronchi M., Petrangolini G., Bosisio S., Allegrini P. (2019). Improved Oral Absorption of Quercetin from Quercetin Phytosome^®^, a New Delivery System Based on Food Grade Lecithin. Eur. J. Drug Metab. Pharmacokinet..

[25] Šebeková K., Brouder Šebeková K. (2019). Glycated Proteins in Nutrition: Friend or Foe?. Exp. Gerontol..

[26] Giacco F., Brownlee M. (2010). Oxidative Stress and Diabetic Complications. Circ. Res..

[27] Srikanth V., Maczurek A., Phan T., Steele M., Westcott B., Juskiw D., Münch G. (2011). Advanced Glycation Endproducts and Their Receptor RAGE in Alzheimer’s Disease. Neurobiol. Aging.

[28] Kim J., Jo Y., Cho D., Ryu D. (2022). L-Threonine Promotes Healthspan by Expediting Ferritin-Dependent Ferroptosis Inhibition in *C. elegans*. Nat. Commun..

[29] Dueñas M., Surco-Laos F., González-Manzano S., González-Paramás A.M., Gómez-Orte E., Cabello J., Santos-Buelga C. (2013). Deglycosylation Is a Key Step in Biotransformation and Lifespan Effects of Quercetin-3-O-Glucoside in *Caenorhabditis elegans*. Pharmacol. Res..

[30] Calabrese E.J. (2004). Hormesis: A Revolution in Toxicology, Risk Assessment and Medicine: Re-framing the Dose–Response Relationship. EMBO Rep..

[31] Back P., Braeckman B.P., Matthijssens F. (2012). ROS in Aging *Caenorhabditis elegans*: Damage or Signaling?. Oxid. Med. Cell. Longev..

[32] Zhou K.I., Pincus Z., Slack F.J. (2011). Longevity and Stress in *Caenorhabditis elegans*. Aging.

[33] Rodriguez M., Snoek L.B., De Bono M., Kammenga J.E. (2013). Worms under Stress: *C. elegans* Stress Response and Its Relevance to Complex Human Disease and Aging. Trends Genet..

[34] Dues D.J., Andrews E.K., Schaar C.E., Bergsma A.L., Senchuk M.M., Van Raamsdonk J.M. (2016). Aging Causes Decreased Resistance to Multiple Stresses and a Failure to Activate Specific Stress Response Pathways. Aging.

[35] Deng J., Dai Y., Tang H., Pang S. (2020). SKN-1 Is a Negative Regulator of DAF-16 and Somatic Stress Resistance in *Caenorhabditis elegans*. G3 Genes Genomes Genet..

[36] Jovic K., Sterken M.G., Grilli J., Bevers R.P.J., Rodriguez M., Riksen J.A.G., Allesina S., Kammenga J.E., Snoek L.B. (2017). Temporal Dynamics of Gene Expression in Heat-Stressed *Caenorhabditis elegans*. PLoS ONE.

[37] Kampkötter A., Timpel C., Zurawski R.F., Ruhl S., Chovolou Y., Proksch P., Wätjen W. (2008). Increase of Stress Resistance and Lifespan of *Caenorhabditis elegans* by Quercetin. Comp. Biochem. Physiol. B Biochem. Mol. Biol..

[38] Das S.S., Sarkar A., Chabattula S.C., Verma P.R.P., Nazir A., Gupta P.K., Ruokolainen J., Kesari K.K., Singh S.K. (2022). Food-Grade Quercetin-Loaded Nanoemulsion Ameliorates Effects Associated with Parkinson’s Disease and Cancer: Studies Employing a Transgenic *C. elegans* Model and Human Cancer Cell Lines. Antioxidants.

[39] Pietsch K., Saul N., Menzel R., Stürzenbaum S.R., Steinberg C.E.W. (2009). Quercetin Mediated Lifespan Extension in *Caenorhabditis elegans* is Modulated by Age-1, Daf-2, Sek-1 and Unc-43. Biogerontology.

[40] Fitzenberger E., Deusing D.J., Marx C., Boll M., Lüersen K., Wenzel U. (2014). The Polyphenol Quercetin Protects The Mev-1 Mutant of *Caenorhabditis elegans* from Glucose-Induced Reduction of Survival under Heat-Stress Depending on SIR-2.1, DAF-12, and Proteasomal Activity. Mol. Nutr. Food Res..

[41] Thériault V., De La Rosa C.M.A., Miard S., Taubert S., Picard F. (2025). Impact of Bacterial Inactivation Methods on *Caenorhabditis elegans* Feeding and Healthspan. Sci. Rep..

[42] Beydoun S., Kitto E.S., Wang E., Huang S., Leiser S.F. (2023). Methodology to Metabolically Inactivate Bacteria for *Caenorhabditis elegans* Research. JoVE.

[43] Han S.K., Lee D., Lee H., Kim D., Son H.G., Yang J.-S., Lee S.-J.V., Kim S. (2016). OASIS 2: Online Application for Survival Analysis 2 with Features for the Analysis of Maximal Lifespan and Healthspan in Aging Research. Oncotarget.

[44] Brinkmann V., Romeo M., Larigot L., Hemmers A., Tschage L., Kleinjohann J., Schiavi A., Steinwachs S., Esser C., Menzel R. (2022). Aryl Hydrocarbon Receptor-Dependent and -Independent Pathways Mediate Curcumin Anti-Aging Effects. Antioxidants.

[45] Diomede L., Romeo M., Rognoni P., Beeg M., Foray C., Ghibaudi E., Palladini G., Cherny R.A., Verga L., Capello G.L. (2017). Cardiac Light Chain Amyloidosis: The Role of Metal Ions in Oxidative Stress and Mitochondrial Damage. Antioxid. Redox Signal..

[46] Komura T., Yamanaka M., Nishimura K., Hara K., Nishikawa Y. (2021). Autofluorescence as a Noninvasive Biomarker of Senescence and Advanced Glycation End Products in *Caenorhabditis elegans*. npj Aging Mech. Dis..

