# Supplementary material for: A Lecithin-Based Delivery Form of Quercetin Promotes Stress Resistance and Longevity in Caenorhabditis elegans
Source: Pharmaceuticals (Basel). 2026 Mar 24;19(4):525. doi: 10.3390/ph19040525 (PMC13118690; doi:10.3390/ph19040525)
Supplement: Supplementary file 1 [file pharmaceuticals-19-00525-s001.zip › pharmaceuticals-4155859-supplementary.pdf]

Supplementary Materials

Article

A lecithin-based delivery form of quercetin promotes stress resistance and longevity in *Caenorhabditis elegans*

Margherita Romeo[1,*], Maria Monica Barzago[1], Claudia Fracasso[1], Manuel Nettis[1], Antonella Riva[2], Marco Gobbi[1], Serena Tongiani[2] and Luisa Diomede[1,*]

[1] Department of Molecular Biochemistry and Pharmacology, Istituto di Ricerche Farmacologiche Mario Negri IRCCS, Via Mario Negri 2, 20156 Milan, Italy; mariamonica.barzago@marionegri.it; claudia.fracasso@marionegri.it; manuel.nettis@marionegri.it; marco.gobbi@marionegri.it

[2] Product Portfolio Office, Indena SpA, 20139 Milan, Italy; riva.antonella@indena.com; tongiani.serena@indena.com

* Correspondence: MR: margherita.romeo@marionegri.it; LD: luisa.diomede@marionegri.it

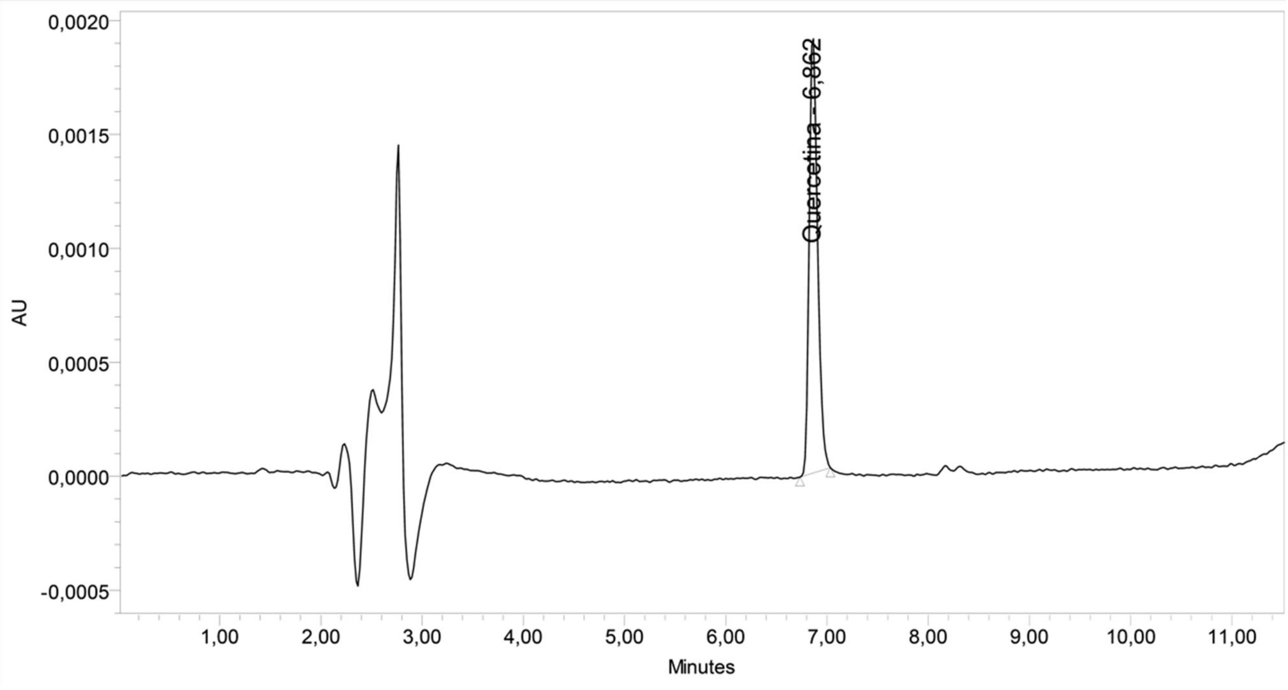

Supplementary Figure 1. Representative chromatogram obtained with a solution of 1 µg/mL quercetin (*i.e.*, the lowest point of the calibration curve).
